# Pretreatment cancer related cognitive impairment and associated psychological factors: a systematic review

**DOI:** 10.3389/fpsyg.2025.1682455

**Published:** 2026-01-07

**Authors:** Aideen Scriney, Lorna Gurren, Pamela Gallagher, Lisa Loughney, Lorraine Boran

**Affiliations:** 1 School of Psychology, Dublin City University, Dublin, Ireland; 2 Department of Medicine, The Royal College of Surgeons in Ireland, Dublin, Ireland

**Keywords:** cancer, pretreatment, cognition, cancer related cognitive impairment (CRCI), anxiety, depression

## Abstract

**Introduction:**

Cancer related cognitive impairment (CRCI) describes issues patients can experience with attention, memory and focus. Understanding the causes of CRCI and the experience of CRCI prior to surgery or treatment is important. Exploring the role of anxiety and depression can aid in understanding how psychological variables may interact with CRCI. Inclusion of both objective CRCI and subjective measures also helps to further understand the relationship between neuropsychological test scores, and self-reported experience. This systematic review was conducted to explore levels of objectively measured and subjectively reported pretreatment CRCI, their inter-relationship, their association with anxiety and depression across cancer types, and changes in CRCI trajectory.

**Methods:**

The review was conducted in line with PRISMA guidelines. Five databases were searched: PsycINFO, CINAHL, MEDLINE, PubMed and EMBASE. Extracted data was narratively synthesised.

**Results:**

Twenty-nine papers remained after full-text screening. Papers varied across cancer types, study design and measurement tools. Sixteen papers included a healthy control (HC) arm. Objective and Subjective CRCI levels were reported both relative to HCs and using scoring criteria or norms. Evidence supported both objective and subjective pretreatment CRCI, but variance increased complexity. Results support relationships between subjective CRCI and psychological variables. Little support was found for a relationship between objective and subjective CRCI. CRCI trajectory across time was explored, but heterogeneity limited further analysis.

**Conclusion:**

Support was found for pretreatment CRCI and relationships between anxiety, depression and subjective cognitive impairment. Variance across study measurement, design and cancer types limited future analysis of variables. Increases in subjective impairment were also observed over time. This review highlights the potential role of psychological factors in pretreatment CRCI, the need for standardization across CRCI research and the importance of control groups as well as norms for analysis to further our understanding of pretreatment CRCI. The findings of this review will help inform clinical care and the development of appropriate interventions for care.

**Systematic review registration:**

https://www.crd.york.ac.uk/PROSPERO/view/CRD42023392837, CRD42023392837.

## Introduction

Cancer related cognitive impairment (CRCI) describes general deficits that some cancer patients may experience in domains including but not limited to memory, attention, processing speed and other executive functions (Lange et al., 2019). It has garnered attention due to its prevalence before, during and after treatment occurs (Hutterer and Oberndorfer, 2021), and due to its association and interaction with overall health related quality of life (Shim et al., 2021), and psychological variables such as anxiety and depression (Hashemi et al., 2020; Brandenbarg et al., 2019; Lange et al., 2019; Hutterer and Oberndorfer, 2021). Indeed, psychological factors have been a particular focus in recent CRCI research, with Haywood and colleagues (Haywood et al., 2024) calling for increased research into the potential transdiagnostic nature of psychopathology and CRCI, and how each concept may functionally contribute and interact with the other into survivorship. Finally, research has focused on the development of guidelines and standardization of CRCI tools and measures, since the establishment of the International Cancer and Cognition Task Force (ICCTF) (Deprez et al., 2018).

Standardization and sensitivity of CRCI measurement has proven challenging. Firstly, traditional neuropsychological tests may not provide adequate sensitivity to measure the mild to moderate impairment experienced by patients with CRCI (Ahles and Hurria, 2018). Secondly, there is a lack of correlation between subjective/self-report of cognitive issues and objective CRCI measurement (Henneghan et al., 2021). CRCI subjective measurement usually involves validated measures like the FACT-cognition (Wagner et al., 2009) or the Cognitive Failures Questionnaire (Broadbent et al., 1982). However, it is also sometimes measured in cancer patients using larger scale quality of life assessments such as the EORTC QLQ-C30 (Aaronson et al., 1993). This can be problematic as the EORTC QLQ-C30 only contains two items that load to the symptom subscale of cognition (Sharma and Brunet, 2023). More work is required to both understand and standardize the process of CRCI measurement (Deprez et al., 2018; Henneghan et al., 2021; Sharma and Brunet, 2023; Wefel et al., 2011), to improve the rigor and reliability of research findings to further our knowledge of CRCI, and to increase the clinical applicability of findings (Henneghan et al., 2021).

Traditionally cognitive impairments related to cancer diagnosis were largely attributed to treatment related factors with terms such as “chemobrain” used to describe the association between the treatment and resulting cognitive deficits (Janelsins et al., 2014; Onzi et al., 2022). Although treatment effects are evident, research has also highlighted the presence of pretreatment CRCI across a wide range of cancer types including breast, leukemia, colorectal cancer and testicular cancers (Olson and Marks, 2019). Notwithstanding difficulties in measuring CRCI, some evidence suggests that pretreatment CRCI can occur in approximately 30% of patients (Janelsins et al., 2014) and is evident from both self-report (11–33%) and neuropsychological (5–11%) tests prior to chemotherapy (Hutterer and Oberndorfer, 2021). The existence and increased evidence of pretreatment CRCI has led researchers to present possible causes of and contributing factors to CRCI beyond treatment related factors, including genetics (Lange et al., 2019; Ahles and Hurria, 2018; Janelsins et al., 2014), inflammatory biomarkers (Lange et al., 2019; Ahles and Hurria, 2018; Janelsins et al., 2014; Andreotti et al., 2015), and tumor derived factors (Ahles and Hurria, 2018; Olson and Marks, 2019; Oppegaard et al., 2023). Additionally, many models and schemas also highlight the role of psychological factors including anxiety, depression, stress and fatigue, as experiencing psychological distress such as anxiety and depression may result in cognitive complaints, as well as the multifaceted nature of fatigue (Lange et al., 2019; Hutterer and Oberndorfer, 2021; Ahles and Hurria, 2018; Andreotti et al., 2015; Oppegaard et al., 2023). More recently, a report on the National Cancer Institute’s meeting on CRCI research design acknowledged the multiple factors at play including cancer, disease burden and treatment, biological mechanisms, patient factors and concurrent symptoms like anxiety and depression (Janelsins et al., 2025). Current clinical guidelines also highlight the potential role of psychological factors, particularly in relation to subjective cognitive function (Zhang et al., 2025), and it is well documented that psychological distress caused by anxiety and depression can impact on cognitive function even in clinically healthy populations (Snyder, 2013).

To date, there have been a number of systematic reviews focusing on the interplay between psychological variables and cancer related cognitive impairment in cancer cohorts undergoing surgery or treatment. Yang and Hendrix (2018) explored psychological factors as they related to CRCI with breast cancer patients. They concluded that depression was most associated with CRCI, followed by anxiety. Oliva et al. (2024) conducted an umbrella review exploring cognitive impairment following breast cancer surgery. Eighteen systematic reviews were included, and they found that while objective and subjective tests were weakly or not correlated, overall CRCI was associated with psychological variables including distress and fatigue. No systematic review has looked at pretreatment CRCI across cancer types or its association with psychological factors. Synthesizing existing evidence would help to elucidate the presence of pretreatment CRCI, and further our understanding of the potential interplay between cognitive function prior to treatment, and baseline anxiety and depression.

To understand pretreatment CRCI and its possible association with anxiety and depression, the aim of this systematic review is to synthesize existing evidence of: (1) Prevalence of pretreatment objective and subjective CRCI (2). Any associations between subjective and objective measures of cognitive function either pretreatment or across time (3). Any associations between pretreatment subjective and objective measures of CRCI and anxiety and/or depression; and (4) Longitudinal changes in CRCI.

## Methods

This review was conducted and reported in line with the preferred reporting for systematic reviews and meta-analyses (PRISMA) statements (Page et al., 2021). The review protocol was registered on the PROSPERO protocol database (No. CRD42023392837).

### Search strategy

**Figure 1:**
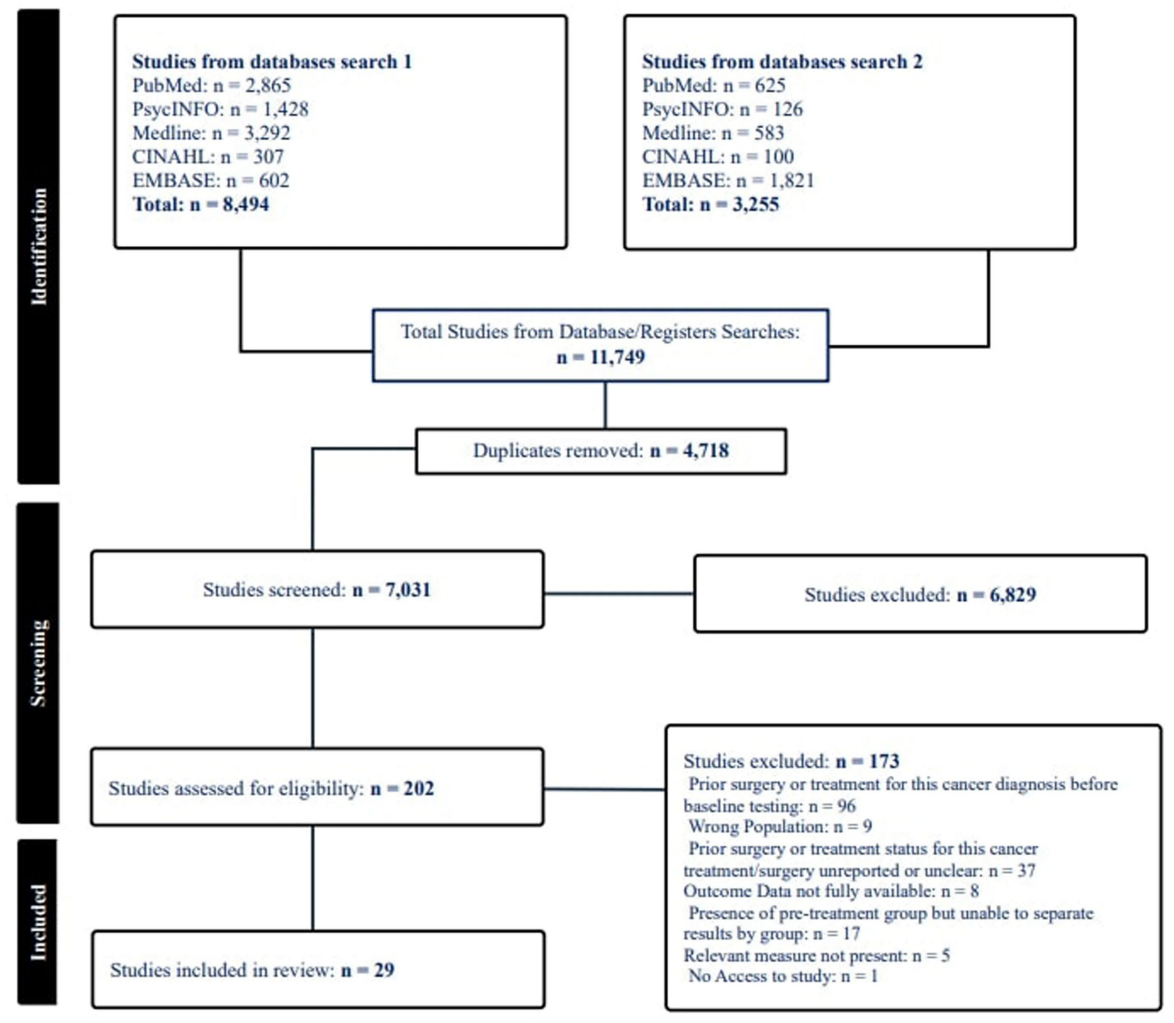
PRISMA flowchart of included studies (*n* = 29).

A systematic literature search of articles relating to CRCI and psychological variables was conducted. Five databases were identified and included based on relevance to the research question: PsycINFO, MEDLINE, CINAHL, EMBASE, and PubMed. Search terms were collated based on relevance to the research question and from previous literature. Search terms were organized into three categories: (1) Cancer Related Terms, (2) Cognition Related Terms, and (3) Psychological Factor Related Terms. Searches were limited to peer reviewed articles published in English with human participants only. Wild card expanders were applied to free text search terms as appropriate for each of the databases. Figure 1 represents the PRISMA screening strategy for each stage of screening. Searches were initially conducted (Search 1) in February 2023 and updated inclusive of January 2025 (Search 2).

### Inclusion criteria

Studies were included if they explored CRCI, and anxiety and/or depression. As the review is focused on the pretreatment time point, data had to be available before any cancer surgery or treatment had occurred. Studies were excluded if participants were <18 years old or were being treated for Central Nervous System (CNS) cancer types, due to the potential associations with cognitive function due to specific cancer type site or tumor location. Full exclusion and inclusion criteria are outlined below in Table 1.

**Table 1:** Inclusion and exclusion criteria.

Inclusion criteria	Exclusion criteria
Explores CRCI Explores anxiety and/or depression	Central nervous system cancers
>18 years of age	Childhood or young adult population
Outcome measurement of cognitive function	Not related to CRCI, anxiety and depression
Outcome Measurement of Anxiety and/or Depression	No pretreatment time point data
Pretreatment and/or surgery time point	No empirical data
Peer reviewed journal articles	Not available in English
Published in English	No measure of cognitive function (subjective or objective) and anxiety and/or depression
Any Study Design	

### Screening process

Screening was conducted in two stages using systematic review software Covidence. Title and abstract and full text screening was conducted alongside a second reviewer (LG). All texts were exported from referencing software Zotero to Covidence where duplicates were then identified and removed. During title and abstract screening, any texts deemed irrelevant by the two reviewers were discarded. This process was repeated for full text screening, where texts were read in full by both reviewers to determine relevance to the research question. Studies were then excluded or included based on specific criteria (see Table 1). If any conflicts or disagreements arose, these were discussed and resolved between the first and second reviewer.

### Quality assessment

Studies included for data extraction were assessed for quality by two reviewers independently (AS and LG) using the Joanna Briggs Institute Critical Appraisal Checklists. Checklists were applied depending on appropriate study methodology with checklists ranging from eight to nine items. These included cohort studies, analytical cross-sectional, case control studies (Moola et al., 2020), randomized control trials (Munn et al., 2020) and prevalence study checklists (Barker et al., 2023). Raters responded to each item using responses “yes,” “no,” “unclear” and “not applicable,” with final assessments categorized as “include,” “exclude” or “seek further information.” The critical appraisal aimed to inform the results of the review by assessing study methodology and the extent to which efforts were made to address potential sources of bias in design. As the purpose of the review was to provide a comprehensive overview of all the available evidence and the quality assessment was undertaken to aid critical evaluation of the findings, the decision was made to not exclude studies on the basis of the quality assessment. After separate quality assessment, the first and second reviewer consulted on any disagreements to reach a consensus. Consensus was reached for all disagreements, so a moderator was not required.

### Data extraction

Data extraction was conducted on eligible studies by the first reviewer. Extracted data included (1) Authors, (2) Study Design, (3) Cancer type and stage, (4) demographic and medical information, (5) Outcomes of interest, (6) Data analysis conducted, (7) Description of Results, and (8) Country. Anxiety and Depression outcome measures were coded as any tool type and associated measurement scale used to measure the construct of anxiety or depression, including also measures with specific subscale scores related to anxiety or depression. Cognitive measures were defined as any objective or subjective measures with the aim of measuring any cognitive construct related to cognitive impairment, performance, function or decline, not screening. This could include cognitive self-report questionnaires or questionnaires containing a distinct cognitive subscale, objective tests of cognitive performance in any domain, or a cognitive screening multidimensional tool.

### Data synthesis

Due to the exploratory nature of this review, included data varied across study designs, measurement tools and cancer types. Due to this, a narrative synthesis was employed to best capture the heterogeneity and variance of studies, whilst also synthesizing results narratively across four main outcomes of interest: (1) Levels of pretreatment objective and subjective CRCI 2. Relationship between objective and subjective CRCI, (3) Relationship between objective and subjective CRCI, and anxiety and depression, (4) Change in cognition over time.

## Results

### Search results

Figure 1 represents the data screening and inclusion process for the current review. Eleven thousand seven hundred forty-nine papers were included for abstract screening. After duplicate removal, 7,031 papers remained. Six thousand eight hundred twenty-nine papers were excluded at the title and abstract screening stage, with 202 papers included at full text screening. After full text screening, 29 papers were included (Aspelund et al., 2024; Bernstein et al., 2018; Cimprich, 1999; Cimprich and Ronis, 2001; ‌Cimprich et al., 2005; ‌Chun et al., 2023; De Rosa et al., 2021; ‌Fayette et al., 2023; François et al., 2024; Gates et al., 2024; ‌Hardy-Leger et al., 2021; Hermelink et al., 2007; Hermelink et al., 2010; Hermelink et al., 2015; ‌Hermelink et al., 2017; ‌Hu et al., 2023; ‌Janelsins et al., 2022; Jung et al., 2022; Juríčková et al., 2023; ‌Kathpalia et al., 2024; ‌Knoerl et al., 2024; Lange et al., 2020; Lehto, 2013; Patel et al., 2015; ‌Patel et al., 2023; ‌Savard et al., 2023; Scheibel, 2004; ‌Tulk et al., 2023; Zhuang et al., 2023). Study characteristics for all papers are included in Table 2.

**Table 2:** Characteristics of included papers.

Author, Year	Country	Cancer type	Study timepoints	Healthy Control Group	Total n, (total, Patients n)	Gender	Average age patients	Cancer related inclusion/Exclusion criteria
Aspelund et al. (2024)	Iceland	Breast	1	Yes	179 (*n* = 112)	F = 179	PG:61.8, CG: 60.9	
Bernstein et al. (2018)	Canada	Head and neck	1	Yes	120 (*n* = 80)	M = 103, F = 17	PG: 58.3, CG: 54.6	Excluded: Metastatic
‌Chun et al. (2023)	South Korea	Small Cell Lung	1	No	21 (*n* = 21)	M = 20, F = 1	Total: 75	
Cimprich (1999)	United States	Breast	1	No	74 (*n* = 74)	F = 74	Total: 56	Included: Stage I-II
Cimprich and Ronis (2001)	United States	Breast	3	Yes	95 (*n* = 47)	F = 95	PG: 64, CG: 61	
Cimprich et al. (2005)	United States	Breast	1	No	184 (*n* = 184)	F = 184	Total 54.6	Included: Stage 0-II
De Rosa et al. (2021)	Italy	Gynaecological	2	No	73 (*n* = 73)	F = 73	Total 50.2	Included: FIGO stage I-III, Excluded: CNS Pathologies
‌Fayette et al. (2023)	Czech Republic	Hodgkin’s lymphoma	3	Yes	81 (*n* = 36)	F = 49, M = 32	PG: 38.63 CG: 41.57	
François et al. (2024)	France	Locally advanced rectal	5	No	101 (*n* = 101)	M = 59, F = 42	Median = 80	Included: Tumor Stage II, III, IV Excluded: Metastatic
Gates et al. (2024)	Australia	Aggressive lymphoma	2	Yes	102 (*n* = 30)	F = 55, M = 47	PG: 57, CG: 56	Excluded: lymphomatous CNS Involvement
‌Hardy-Leger et al. (2021) *	France	Breast	1	No	264	F = 264 (263 in analysis)	Total: 54.0	Included: cT0-cT3, cN0-3 tumor
‌Hermelink et al. (2007)**	Germany	Breast	2	No	109 (*n* = 109)	F = 109, (101 in analysis)	Total: 48.6	Included: Stage 0-IIIc Excluded Metastasis
Hermelink et al. (2010)**	Germany	Breast	3	No	101 (*n* = 101)	F = 101	Total: 48.5	Included: Stage 0-IIIc Excluded Metastasis
Hermelink et al. (2015)†	Germany	Breast	1	Yes	226 (*n* = 60)	F = 226	PG: 50.4, CG: 52.6	Included: Stage 0-IIIc Excluded: Metastasis
‌Hermelink et al. (2017)†	Germany	Breast	3	Yes	206 (*n* = 150)	F = 206	Patient Chemotherapy: m = 47.7 Patient Non-Chemotherapy: m = 53.4, Controls: m = 52.3	Included Stage 0-IIIc Excluded: Metastasis
‌Hu et al. (2023)	China	Breast	3	Yes	74 (*n* = 36)	F = 74	PG:47.9, CG 1: 52.9, CG 2: 44.94	Excluded: Presence of brain tumor
Janelsins et al. (2022)	United States	Lymphoma	3	Yes	460 (*n* = 248)	F = 180, M = 280	PG: 55.40, CG: 53.96	Excluded: CNS disease
Jung et al. (2022)	South Korea	Thyroid	1	No	130 (*n* = 130)	F = 130 (124 in analysis)	Total: 43.99	Excluded: metastasis
Juríčková et al. (2023)	Czech Republic	Hodgkin Lymphoma	1	Yes	80 (*n* = 40)	F = 44, M = 36	PG: 39.14, CG: 38.41	
Kathpalia et al. (2024)	India	Non-Hodgkin Lymphoma	1	Yes	62 (*n* = 31)	F = 27, M = 35	PG: 51.9, CG: 50	Excluded: Presence of secondary cancer
‌Knoerl et al. (2024)	United States	Breast	2	No	49 (*n* = 49)	F = 49 (47 in analysis)	Total: 52.8	Included: Stage I-III
Lange et al. (2020)*	France	Breast	1	Yes	396 (*n* = 264)	F = 396	PG: 54.1, CG: 53.2	Included: cT0-cT3, cN0-3 tumor
Lehto (2013)	United States	Lung Malignancy	2	Yes	23 (*n* = 15)	F = 8, M = 15	Total: 61.4	
Patel et al. (2015) † †	United States	Breast	1	Yes	262 (*n* = 174)	F = 262	PG: 61.82, CG: 60.48	Excluded: Metastasis
Patel et al. (2023) † †	United States	Breast	4	Yes	250 (*n* = 173)	F = 250	PG: 60, CG: 61	Excluded: Metastasis
‌Savard et al. (2023)	Canada	Prostate	4	No	130 (*n* = 130)	M = 130	Total: 63.39	
Scheibel (2004)	United States	Myelogenous leukemia	2	No	30 (*n* = 30)	F = 10, M = 20	PG: 46.0	
‌Tulk et al. (2023)	Canada	Prostate	2	No	24 (*n* = 24)	M = 24	Total 69.63	Included: T2,3,4
Zhuang et al. (2023)	China	Breast	2	Yes	41 (*n* = 21)	F = 41	PG 44 CG 40.3	Included: Stage I-III Excluded Metastasis

* = Papers contained data from the same patient cohort, ** = Papers contained data from the same patient cohort, † = Papers contained data from the same patient cohort, †† = Papers contained data from the same patient cohort, PG = Patient Group, CG = Control Group.

### Quality assessment

Although studies were not dependent on quality assessment for inclusion, study quality was generally high, and all included studies were deemed adequate for inclusion by both reviewers. The full quality assessment is included in Supplementary material 1.

### Characteristics of included papers

Study characteristics of included papers are presented below (Table 2). Papers included the data of 2,920 patients. Patient sample size ranged from 15 (Lehto, 2013) to 264 (Lange et al., 2020) patients. The average patient age was 55.09 years. Sixteen papers had female only participants (Cimprich, 1999; Cimprich and Ronis, 2001; ‌Cimprich et al., 2005; De Rosa et al., 2021; ‌Hardy-Leger et al., 2021; Hermelink et al., 2007; Hermelink et al., 2010; Hermelink et al., 2015; ‌Hermelink et al., 2017; ‌Hu et al., 2023; Jung et al., 2022; ‌Knoerl et al., 2024; Lange et al., 2020; Patel et al., 2015; ‌Patel et al., 2023; Zhuang et al., 2023) (Table 2). Publication dates ranged from 1999 to 2024, with 18 papers published within the last 5 years (See Table 2). Eleven papers were cross sectional in design (Aspelund et al., 2024; Bernstein et al., 2018; Cimprich, 1999; ‌Cimprich et al., 2005; ‌Hardy-Leger et al., 2021; Hermelink et al., 2015; Jung et al., 2022; Juríčková et al., 2023; ‌Kathpalia et al., 2024; Lange et al., 2020; Patel et al., 2015), one was retrospective (‌Chun et al., 2023) while 17 papers had prospective and/or longitudinal designs with multiple assessment timepoints (Cimprich and Ronis, 2001; ‌Cimprich et al., 2005; ‌Chun et al., 2023; De Rosa et al., 2021; ‌Fayette et al., 2023; François et al., 2024; Gates et al., 2024; ‌Hardy-Leger et al., 2021; Hermelink et al., 2007; Hermelink et al., 2010; ‌Hermelink et al., 2017; ‌Hu et al., 2023; ‌Janelsins et al., 2022; ‌Knoerl et al., 2024; Lehto, 2013; ‌Patel et al., 2023; ‌Savard et al., 2023; Scheibel, 2004; ‌Tulk et al., 2023; Zhuang et al., 2023). Sixteen papers included a healthy control group for analysis (Table 2) (Aspelund et al., 2024; Bernstein et al., 2018; Cimprich and Ronis, 2001; ‌Fayette et al., 2023; Gates et al., 2024; Hermelink et al., 2015; ‌Hermelink et al., 2017; ‌Hu et al., 2023; ‌Janelsins et al., 2022; Juríčková et al., 2023; ‌Kathpalia et al., 2024; Lange et al., 2020; Lehto, 2013; Patel et al., 2015; ‌Patel et al., 2023; Zhuang et al., 2023).

Cancer type varied across papers, but included 15 breast cancer papers (Aspelund et al., 2024; Cimprich, 1999; Cimprich and Ronis, 2001; ‌Cimprich et al., 2005; ‌Hardy-Leger et al., 2021; Hermelink et al., 2007; Hermelink et al., 2010; Hermelink et al., 2015; ‌Hermelink et al., 2017; ‌Hu et al., 2023; ‌Knoerl et al., 2024; Lange et al., 2020; Patel et al., 2015; ‌Patel et al., 2023; Zhuang et al., 2023). As shown in Table 2, Thirteen papers applied inclusion criteria based on cancer/tumor stage (Cimprich, 1999; ‌Cimprich et al., 2005; De Rosa et al., 2021; François et al., 2024; ‌Hardy-Leger et al., 2021; Hermelink et al., 2007; Hermelink et al., 2010; Hermelink et al., 2015; ‌Hermelink et al., 2017; ‌Knoerl et al., 2024; Lange et al., 2020; ‌Tulk et al., 2023; Zhuang et al., 2023). Fifteen papers excluded participants based on presence of metastasis or CNS disease (Bernstein et al., 2018; De Rosa et al., 2021; François et al., 2024; Gates et al., 2024; Hermelink et al., 2007; Hermelink et al., 2010; Hermelink et al., 2015; ‌Hermelink et al., 2017; ‌Hu et al., 2023; ‌Janelsins et al., 2022; Jung et al., 2022; ‌Kathpalia et al., 2024; Patel et al., 2015; ‌Patel et al., 2023; Zhuang et al., 2023). With regard to treatment type, eight papers explored surgery (Aspelund et al., 2024; Cimprich, 1999; Cimprich and Ronis, 2001; ‌Cimprich et al., 2005; Jung et al., 2022; ‌Knoerl et al., 2024; Lehto, 2013; ‌Savard et al., 2023), eight chemotherapy only (‌Fayette et al., 2023; Gates et al., 2024; Hermelink et al., 2007; Hermelink et al., 2010; ‌Hu et al., 2023; ‌Janelsins et al., 2022; Scheibel, 2004; Zhuang et al., 2023), and the remaining 13 papers explored combinations of treatment types (Bernstein et al., 2018; ‌Chun et al., 2023; De Rosa et al., 2021; François et al., 2024; ‌Hardy-Leger et al., 2021; Hermelink et al., 2015; ‌Hermelink et al., 2017; Juríčková et al., 2023; ‌Kathpalia et al., 2024; Lange et al., 2020; Patel et al., 2015; ‌Patel et al., 2023; ‌Tulk et al., 2023).

### Included measures

Measures included in each paper are outlined below in Table 3. Fifteen papers measured both objective and subjective cognitive function (Aspelund et al., 2024; Bernstein et al., 2018; ‌Cimprich et al., 2005; François et al., 2024; Gates et al., 2024; ‌Hardy-Leger et al., 2021; Hermelink et al., 2007; Hermelink et al., 2010; Hermelink et al., 2015; ‌Hermelink et al., 2017; ‌Hu et al., 2023; ‌Janelsins et al., 2022; Lange et al., 2020; ‌Patel et al., 2023; ‌Tulk et al., 2023). Nine papers measured objective impairment only (Cimprich and Ronis, 2001; ‌Chun et al., 2023; ‌Fayette et al., 2023; Jung et al., 2022; Juríčková et al., 2023; ‌Kathpalia et al., 2024; Patel et al., 2015; Scheibel, 2004; Zhuang et al., 2023). Five papers measured subjective impairment only (Cimprich, 1999; De Rosa et al., 2021; ‌Knoerl et al., 2024; Lehto, 2013; ‌Savard et al., 2023).

**Table 3:** Characteristics of measures.

Author	Ob.	Sub.	Objective Cognitive measures	Reported Cognitive Domains	Sub. measures	Anxiety	Depression
Aspelund et al. (2024)	X	X	5 s psychomotor visual test, TMT A&B, digit span, RVALT, COWA test.	Attention, executive function, processing speed, working and verbal memory, verbal fluency	PROMIS CF 8a	GAD-7	CES-D
Bernstein et al. (2018)	X	X	Vocabulary and matrix reasoning, digit span, spatial span, HVLT-R, brief visuospatial memory test -R, the D-KEFS Stroop test, TMT A&B, grooved pegboard	Concentration/ attention, executive function, memory, motor dexterity processing speed	Fact Cog V3	HADS	HADS
‌Chun et al. (2023)	X		MMSE	Cognitive function			GDSSF-K
Cimprich (1999)		X			AFI	POMS	POMS
Cimprich and Ronis (2001)	X		Digit span, digit symbol, the Necker cube pattern control test	Capacity to direct attention			POMS
Cimprich et al. (2005)	X	X	Digit span, TMT A&B, three shapes three words test	Capacity to direct attention, short-term memory	AFI	POMS-SF	POMS-SF
De Rosa et al. (2021)		X			Fact Cog V3		BDI
‌Fayette et al. (2023)	X		Auditory verbal learning test, complex figure test (ROCFT), TMT A&B, verbal fluency test, logical memory test, continuous performance test 3, digit span, similarities, digit symbol, letter number sequencing, the Stroop test.	Abstraction/executive functions, attention/vigilance, verbal memory and learning, working memory/flexibility, processing speed/psychomotor speed		HAM-A BAI	HAM-D BDI
François et al. (2024)	X	X	MMSE	Attention and calculation, language, memory recall, orientation, registration, visuospatial ability	EORTC-CF		GDS
Gates et al. (2024)	X	X	TMT A&B, HVLT-R, digit span, the Stroop test, COWA test	Attention, executive function, learning and memory, processing speed, verbal fluency, working memory	FACT-Cog V3	PROMIS-7a	PROMIS-8b SF
‌Hardy-Leger et al. (2021)	X	X	HVLT, digit span, letter-number sequencing, spatial span, TMT A&B, symbol search, the Stroop test, D2 test, Fluency score	Attention, executive function, episodic memory, processing speed, working memory	FACT-Cog V3	HADS	HADS
Hermelink et al. (2007)	X	X	Logical memory I, II, D2 test, digit symbol, TMT A&B, Regensburg word fluency test, digit span	Attention, concentration, cognitive flexibility, divided attention, executive function, processing speed, psychomotor function, selective attention, verbal memory, verbal working memory	EORTC-CF, FEDA	HADS	HADS
Hermelink et al. (2010)	X	X	Logical memory I, II, D2 test, digit symbol test, TMT A&B, Regensburg word fluency test, digit span	Attention, concentration, cognitive flexibility, divided attention, executive function, processing speed, psychomotor function, selective attention, verbal memory, verbal working memory	EORTC-CF, FEDA	HADS	HADS
Hermelink et al. (2015)	X	X	Go/Nogo, TMT A&B, test of attentional performance, digit span, verbal learning and memory test, R-Word fluency test,	Attention, executive function, memory, processing speed, verbal memory	EORTC-CF, FEDA		PHQ-D
Hermelink et al. (2017)	X	X	Go/Nogo, TMT A&B, test of attentional performance, digit span, verbal learning and memory test, R-Word fluency test,	Attention, executive function, memory, processing speed, verbal memory	EORTC-CF, FEDA		PHQ-D
‌Hu et al. (2023)	X	X	TMT A, VFT, digit span	Attention, executive function, motor skills, processing speed, working memory, semantic memory, verbal fluency, visual search	FACT-Cog	SAS	SDS
Janelsins et al. (2022)	X	X	CANTAB, Hopkins verbal learning & memory test, TMT A&B, COWA	Attention, executive function, memory	FACT-Cog	STAI	MFSI
Jung et al. (2022)	X		Digit span, COWA, TMT A&B, the Stroop test	Attention, cognitive control (Executive Function)			PHQ-8
Juríčková et al. (2023)	X		Auditory verbal learning Test, Rey-Osterrieth complex figure test, TMT A&B, VFT, Continuous performance test, WAIS-III: Digits span, similarities, digit symbols- coding, letter number sequencing. Logical memory from Wechsler memory (WMS-IIIa) subtest and the Stroop test.	Abstraction/ executive functions, attention/vigilance, memory and learning, processing speed /psychomotor speed, verbal memory and learning, working memory/flexibility		HAM-A, BAI	HAM-D, BDI
Kathpalia et al. (2024)	X		MoCA	Attention and concentration, executive function, calculations and orientation, conceptual thinking, language, memory, visuo-constructional skills			PHQ-9
‌Knoerl et al. (2024)		X			EORTC-CF	HADS	HADS
Lange et al. (2020)	X	X	HVLT, digit span, letter-number sequencing, spatial span, TMT A&B, symbol search, the Stroop test, D2 test, fluency score	Attention and executive function, episodic memory, processing speed, verbal fluency, working memory	FACT-Cog	HADS	HADS
Lehto (2013)		X			AFI	PSWQ	
Patel et al. (2015)	X		Trials 4, color-word inhibition, inhibition switching from the D-KEFS, HVLT (Total and delayed recall, verbal mem), processing speed index -WAIS-IV	Executive function, memory, processing speed		BSI	
Patel et al. (2023)	X	X	Wechsler digit span scale	Attention/concentration (working memory)	BRIEF-A		BSI 18
‌Savard et al. (2023)		X			Fact-Cog	HADS	HADS-PHQ-9
Scheibel (2004)	X		Digit symbol, Consistent long-term retrieval and 30 min delayed recall from VSRT, TMT A&B, COWA test	Graph-motor speed, verbal learning, verbal memory, verbal fluency, visual-motor and sequencing skills			MMPI
‌Tulk et al. (2023)	X	X	HVLT-R, COWA test, letter number sequencing	Lexical fluency, verbal learning and memory, working memory	FACT-Cog	HADS	HADS
Zhuang et al. (2023)	X		MMSE, number connection test, digit symbol, line tracing test, serial dot test, the Stroop test, auditory verbal learning test,	Attention, executive function, fine motor skills, long-term memory, processing speed, short-term memory, visual ability, reaction capability		SAS	SDS

TMT = Trail Making Test, RVALT = Rey Auditory Verbal Learning Fluency Test; COWA = Controlled Oral Word Association, HVLT-(R) = Hopkins Verbal Learning Test-(Revised), D-KEFS = Delis-Kaplan Executive Function System, MMSE= Mini Mental State Examination, VFT = Verbal Fluency Test, DST = Digit Span Test, CANTAB = Cambridge Neuropsychological Test Automated Battery, WMS = Wechsler Memory Scale, MoCa = Montreal Cognitive Assessment, WAIS = Wechsler Adult Intelligence Scale, VSRT = Verbal Selective Reminding Test, PROMIS CF-8a = Patient-Reported Outcomes Measurement Information System 8a, FACT-Cog = Functional Assessment of Cancer Therapy, EORTC-CF = European Organisation for Research and Treatment of Cancer- Cognitive Function scale, FEDA = Fragebogen erlebter Defizite der Aufmerksamkeit (The Questionnaire of Experienced Deficits of Attention), BRIEF-A = Behavior Rating Inventory of Executive Function-Adult Version.GAD-7 = Generalized Anxiety Disorder-7, CES-D = Centre for Epidemiological Studies Depression Scale, GDS SF-K = Geriatric Depression Scale Short Form -Korean, GDS = Geriatric Depression Scale, HADS = Hospital Anxiety and Depression Scale, POMS = Profile of Mood States, POMS-SF = Profile of Mood States- Short Form, BDI = Beck Depression Inventory, BAI = Beck Anxiety Inventory, HAM-A = Hamilton Anxiety, HAM-D = Hamilton Depression, PHQ-D = Patient Health Questionnaire- Depression, STAI = Spielberger Trait Anxiety Inventory, MFSI = Multidimensional Fatigue Symptom Inventory, PHQ-8 = Patient Health Questionnaire -8, PHQ-9 = Patient Health Questionnaire-9, PSWQ = Penn State Worry Questionnaire, PROMIS = Patient-Reported Outcomes Measurement Information System, PROMIS 8b SF = Patient-Reported Outcomes Measurement Information System, 8b Short Form, BDI-II = Beck Depression Inventory-II, BSI = Brief Symptom Inventory, BSI-18 = Brief Symptom Inventory-18, MMPI = Minnesota Multiphasic Personality Inventory, SAS = Self-rating Anxiety Scale, SDS = Self-rating Depression Scale.

Twenty-four papers explored cognitive impairment using objective cognitive neuropsychological assessments (see Table 3). The most frequent tests included were the trail making test (*n* = 16), Digit span (*n* = 16) and the Stroop color-word test (*n* = 8), followed by the Hopkins verbal learning test-revised (*n* = 7), the digit symbols test (*n* = 7), and the controlled oral word association test (*n* = 6). The most common domains measured across papers were attention (*n* = 20), memory (*n* = 20), and executive functions (*n* = 18) (see Table 3). Twenty papers explored CRCI using subjective measures including The Functional Assessment of Cancer Therapy (FACT) Cognition scale (*n* = 9), The Attentional Function Index (*n* = 3), the cognitive function subscale of the European Organization for Research and Treatment of Cancer questionnaire (EORTC) (*n* = 6), the cognitive function subscale of the Patient-Reported Outcomes Measurement Information System (PROMIS) (*n* = 1), The Questionnaire of Experienced Attention Deficits (FEDA) (*n* = 4) and the Brief Rating Inventory of Executive Function-Adult (BRIEF-A) (*n* = 1) (see Table 3).

For psychological outcomes, eight papers included measures of depression only (Cimprich and Ronis, 2001; ‌Chun et al., 2023; De Rosa et al., 2021; François et al., 2024; Jung et al., 2022; ‌Kathpalia et al., 2024; ‌Patel et al., 2023; Scheibel, 2004), two papers included measures of anxiety only (Lehto, 2013; Patel et al., 2015), The remaining 19 papers included a measure of both anxiety and depression (Aspelund et al., 2024; Bernstein et al., 2018; Cimprich, 1999; ‌Cimprich et al., 2005; ‌Fayette et al., 2023; Gates et al., 2024; ‌Hardy-Leger et al., 2021; Hermelink et al., 2007; Hermelink et al., 2010; Hermelink et al., 2015; ‌Hermelink et al., 2017; ‌Hu et al., 2023; ‌Janelsins et al., 2022; Juríčková et al., 2023; ‌Knoerl et al., 2024; Lange et al., 2020; ‌Savard et al., 2023; ‌Tulk et al., 2023; Zhuang et al., 2023) (See Table 3).

### Level of pretreatment cognitive impairment measured by objective and subjective assessments

Within the 29 papers included in analysis, the data of 25 discrete studies was reported. Three papers (Hermelink et al., 2010; ‌Hermelink et al., 2017; ‌Patel et al., 2023) reported on follow-up timepoints for the same patient cohort as (Hermelink et al., 2007; Hermelink et al., 2015; Patel et al., 2015) respectively. One study, ‌Hardy-Leger et al. (2021) conducted additional analyses on the same patient cohort as Lange et al. (2020). Studies varied in the ways they chose to measure levels of objective or subjective cognitive impairment. This can be distilled into two main measurement approaches: (1) Comparison with healthy control group, (2) Predefined test criteria. A common predefined criteria was use of the International Cancer and Cognition Taskforce criteria for objective cognitive impairment. This was proposed as a criteria of a mean score ≤1.5 standard deviations below healthy controls or norms on at least two tests or one test with a mean score standard deviation of ≤2.0 (Wefel et al., 2011). Other common approaches included standardized tests, population or age adjusted norms for a given test, or standard cut-offs for a given subjective cognitive measure.

#### Objective cognitive impairment Pretreatment

Twenty studies explored objective cognitive impairment at the pretreatment timepoint. Results are summarized below in Table 4. Impairment was recorded either by test, as a global cognitive function score, or by cognitive domain. Impairment was defined as both relative to healthy control groups and using predefined test norms. Fourteen studies included a healthy control group (HCs) alongside patient groups (PGs) (Table 4). Ten of these 14 studies reported significant results on at least one test compared to HCs (Aspelund et al., 2024; Cimprich and Ronis, 2001; ‌Fayette et al., 2023; Gates et al., 2024; Hermelink et al., 2015; ‌Hu et al., 2023; Juríčková et al., 2023; ‌Kathpalia et al., 2024; Lange et al., 2020; Patel et al., 2015). For most studies, this was in the direction of reduced performance compared to HCs. One study reported better performance on one test for PGs compared to HCs (‌Hu et al., 2023), while two studies reported increased performance compared to norms for PGs compared to norms on some test indices (Hermelink et al., 2007; Hermelink et al., 2015). The most common reported domains were processing speed and executive functions. Eleven studies reported levels of cognitive impairment within patient groups applying population norms or predefined test criteria. Within these, reported observed impairment in at least one domain ranged from 13.8% (Aspelund et al., 2024) to 78.2% (Jung et al., 2022).

**Table 4:** Summary of findings for pretreatment objective cognitive impairment.

Study	Patient group (PG) comparisons to healthy control (HC) groups findings			Patient Groups (PG) based on impairment criteria or norms	
	HC Group Y/N	Differences PGs and HCs Y/N	Findings	PGs Test Norms Y/N	Findings
Aspelund et al. (2024)	Y	Y	PG Sig. worse processing speed (*p* = 0.01) PG Sig. worse verbal memory (*p* < 0.001) Overall prevalence of CI non sig	Y	13.8% prevalence of CI
Bernstein et al. (2018)	Y	N	No Sig. Differences	Y	11.3% impaired in 2 or more domains 33.8% impaired in one domain
‌Chun et al. (2023)	N			Y	Scores within test norms
Cimprich and Ronis (2001)	Y	Y	PG sig. Worse scores compared to HCs (*p* < 0.05)	Y	Scores fell within test norms
Cimprich et al. (2005)	N			Y	Scores fell within test norms
‌Fayette et al. (2023)	Y	Y	PG Sig. Worse verbal memory and learning, speed of processing/psychomotor speed and abstraction/executive functions	N	
François et al. (2024)	N			Y	Scores fell within test norms
Gates et al. (2024)	Y	Y	PG Sig. worse on average for all neuropsychological tests. (all *p* ≤ 0.033)	N	
Hermelink et al. (2007, 2010)	N			Y	5 tests showed sig. Worse scores than norms 1 test showed sig. Better (digit symbol) 31% patients ≥ 2 tests in the lower 5% range. 32% patients had moderate CI
Hermelink et al. (2015, 2017)	Y	Y	PGs sig worse behavioral control indices go/no commission and omission errors. All other indices non sig.	Y	PGs Sig. worse on seven indices PGs Better score on three indices All case patients 41.3% prevalence based on the least stringent definition of impairment. 8% based on most stringent.
‌Hu et al. (2023)	Y	Y	PGs Sig. worse verbal fluency PGs Sig worse digit span PGs Sig better Trail making Test (A)	N	
Janelsins et al. (2022)	Y	N	No Sig differences	N	
Jung et al. (2022)	N			Y	78.2% of patients impaired based on applied criteria. Working memory domain most common.
Juríčková et al. (2023)	Y	Y	PGs Sig. worse Auditory Verbal Learning Test PGs Sig. worse Verbal Fluency Test PGs Sig. worse Trail Making Test (A&B)	N	
Kathpalia et al. (2024)	Y	Y	PGs Sig worse cognitive function (MoCa) (*p* < 0.001) PGs Sig worse MoCa subdomains attention, concentration and calculation, language, memory, conceptual thinking (*p* = 0.001-p < 0.05)	N	
Lange et al. (2020) and ‌Hardy-Leger et al. (2021)	Y	Y	PGs Sig worse overall cog impairment and all subdomains attention, EF, WM, Processing speed (p < 0.001)	Y	CI observed in 28% of patients
Patel et al. (2023, 2015)	Y	Y	PGs Sig. worse Executive function, processing speed, verbal memory (*p* = 0.02-*p* < 0.001) PGs worse attention/concentration *p* < 0.05	N	
Scheibel (2004)	N			Y	Digit symbol scores fell within norms
‌Tulk et al. (2023)	N			Y	CI observed in 29% of patients
Zhuang et al. (2023)	Y	N	No difference between PGs and HCs	N	Scores fell within test cut off

#### Subjective impairment pretreatment

Seventeen studies explored subjective cognitive impairment at the pretreatment timepoint. Results are summarized below in Table 5. For the given subjective measures, a higher score indicates better perceived cognitive function. Impairment was defined as both relative to healthy control groups and using measure scoring criteria. Nine of the 17 studies included a healthy control group (HCs) alongside patient groups (PGs) (Table 5). With regards to healthy control groups, 7 of the 9 studies reported worse performance of patient groups compared to healthy controls (Aspelund et al., 2024; Bernstein et al., 2018; Gates et al., 2024; Hermelink et al., 2015; ‌Janelsins et al., 2022; Lange et al., 2020; Lehto, 2013). Nine studies reported scores or levels of perceived impairment for patient groups using predefined criteria or norms. Across these studies reported prevalence for subjective measures ranged from 17.55% (‌Savard et al., 2023) to 29% (‌Tulk et al., 2023) (see Table 5).

**Table 5:** Summary of findings for pretreatment subjective cognitive impairment.

Study	HC findings			PGs based	
	HC Group Y/N	Differences Y/N	Findings	PG Measure Scores Y/N	Findings
Aspelund et al. (2024)	Y	Y	PG Sig. Worse Cog complaints on PROMIS-CF *p* < 0.001	N	
Bernstein et al. (2018)	Y	Y	PG Sig. Worse FACT-Cog Subscales (unadjusted *p* < 0.001–0.012)	N	
Cimprich (1999)	N			Y	Only 27% of patients in highest interquartile range of AFI
Cimprich et al. (2005)	N			Y	25% of PG effective cognitive functioning on AFI 50% moderate cognitive function on AFI 25% lowest level of cognitive function on AFI
De Rosa et al. (2021)	N			Y	PGs FACT-Cog Perceived Cog Impairment Subscale High score (61.35/72)
François et al. (2024)	N			Y	PGs EORTC-CF 86.43/100
Gates et al. (2024)	Y	Y	Sig. effect of group on FACT-Cog perceived cognitive impairments impact on QoL	N	
‌Hardy-Leger et al. (2021) and Lange et al. (2020)	Y	Y	PGs sig worse scores on FACT-Cog *p* < 0.01	Y	15% of patients reported significant complaints in both Perceived cognitive impairment (PCI) and cognitive abilities domains. 2% in all domains. 24.3% PCI domain only.
Hermelink et al. (2007, 2010)	N			Y	PGs EORTC-CF Score 84/100 PGs FEDA Score 89.9/108
Hermelink et al. (2015, 2017)	Y	Y	PGs Sig Worse score on FEDA and EORTC-CF PGs Sig Worse score on EORTC-CF Case Matched	N	
‌Hu et al. (2023)	Y	N	No Sig. differences	N	
Janelsins et al. (2022)	Y	Y	PGs sig worse scores on FACT-Cog (*p* = 0.01) PGs sig worse scores on self-rated attention (*p* = 01)	N	
‌Knoerl et al. (2024)	N			Y	PGs EORTC-CF Score high (77/100)
Lehto (2013)	Y	Y	PGs Worse scores on AFI	N	
Patel et al. (2023)	Y	N	No. Sig Differences	N	
‌Savard et al. (2023)	N			Y	PGs 17.55% FACT-Cog Perceived Cognitive Impairment Subscale (<54)
‌Tulk et al. (2023)	N			Y	PGs 29% Prevalence FACT-Cog

### Relationships between objective and subjective cognitive impairment

Twelve studies included a measure of both objective and subjective cognitive function (see Table 3). Of these, five studies did not directly explore the relationship between objective and subjective measurement (Aspelund et al., 2024; François et al., 2024; ‌Hu et al., 2023; ‌Janelsins et al., 2022; ‌Patel et al., 2023). Objective measures included associations between subjective cognitive impairment and individual tests, specific domains, or composite general cognitive function scores. Overall, seven of the 12 studies reported on associations between objective and subjective measures of cognitive function across nine individual papers (Bernstein et al., 2018; ‌Cimprich et al., 2005; ‌Hardy-Leger et al., 2021; Hermelink et al., 2007; Hermelink et al., 2010; Hermelink et al., 2015; ‌Hermelink et al., 2017; Gates et al., 2024; ‌Tulk et al., 2023).

Two studies found some associations between subjective and objective cognitive function. Bernstein and colleagues (Bernstein et al., 2018) reported that all objective tests other than motor dexterity correlated with “comments from others” and “perceived cognitive abilities” subscales of the Fact-Cog in Supplementary material. Similarly, Hermelink et al. (2015) reported a significant correlation between omission errors in the go/no go cognitive test and scores on self-reported cognitive function measured by the FEDA and EORTC. At one year post chemotherapy, ‌Hermelink et al. (2017), reported in their follow up paper that scores on the FEDA measure of subjective cognitive function correlated significantly with the composite score of cognitive performance.

The remaining five studies reported no associations between objective and subjective measures of cognitive function, either as correlations (‌Cimprich et al., 2005; Hermelink et al., 2007; Gates et al., 2024), as a function of change across time (Hermelink et al., 2010; ‌Tulk et al., 2023), or in multivariate analysis (Lange et al., 2020). Interestingly, a paper by ‌Hardy-Leger et al. (2021), conducted a sub analysis on the same data set as Lange et al. (2020) by grouping patients based on degree of subjective cognitive complaints, ranging from “no complaints” to “consistent complaints” across five groups. They found that the no complaints group performed significantly better in executive function tasks than the significant complaints groups.

### Relationship between cognition and psychological variables

Nineteen studies explored the relationship between anxiety and/or depression and cognitive function (Aspelund et al., 2024; Bernstein et al., 2018; Cimprich, 1999; Cimprich and Ronis, 2001; ‌Cimprich et al., 2005; ‌Fayette et al., 2023; Gates et al., 2024; Hermelink et al., 2007; Hermelink et al., 2015; ‌Janelsins et al., 2022; Jung et al., 2022; Juríčková et al., 2023; ‌Knoerl et al., 2024; Lange et al., 2020; Lehto, 2013; Patel et al., 2015; ‌Savard et al., 2023; Scheibel, 2004; ‌Tulk et al., 2023). Thirteen studies included both anxiety and depression (Aspelund et al., 2024; Bernstein et al., 2018; Cimprich, 1999; ‌Cimprich et al., 2005; ‌Fayette et al., 2023; Gates et al., 2024; Hermelink et al., 2007; ‌Janelsins et al., 2022; Juríčková et al., 2023; ‌Knoerl et al., 2024; Lange et al., 2020; ‌Savard et al., 2023; ‌Tulk et al., 2023).

#### Anxiety

Eleven studies reported on the relationship between objective cognitive function and anxiety (Table 6). Of these, eight studies reported no relationship between anxiety and objective cognitive function (Aspelund et al., 2024; ‌Fayette et al., 2023; Gates et al., 2024; Hermelink et al., 2015; Juríčková et al., 2023; Lange et al., 2020; Patel et al., 2015; ‌Tulk et al., 2023). Three studies reported significant associations between at least one objective cognitive test and measures of anxiety (Bernstein et al., 2018; ‌Cimprich et al., 2005; ‌Janelsins et al., 2022). Based on reported domains (Table 3), represented domains included capacity to direct attention (‌Cimprich et al., 2005), attention and concentration, processing speed, motor dexterity (Bernstein et al., 2018), memory, and executive function (‌Janelsins et al., 2022). In most cases, the relationships indicated that increased anxiety was associated with increased subjective cognitive impairment and decreased objective cognitive performance, except for one objective (‌Cimprich et al., 2005) report. Ten studies reported on the relationship between anxiety and subjective cognitive function (Table 6). Of these, two studies (Aspelund et al., 2024; Lange et al., 2020) reported a non-significant relationship between self-reported cognitive impairment and anxiety. Relationships are summarized in Table 6 below.

**Table 6:** Summary of relationships between anxiety and cognitive impairment.

Study	Objective cognitive impairment			Subjective cognitive impairment	
	Analysis	Significant relationship Y/N	Findings	Significant relationship Y/N	Findings
Aspelund et al. (2024)	Regression	N	No relationship between objective tests and Anxiety	N	No Sig. relationship between subjective cognitive impairment and Anxiety
Bernstein et al. (2018)	Correlations	Y	Sig. Inverse correlation between motor dexterity, processing speed and concentration with HADS Anxiety Scores	Y	Sig. Inverse correlation between FACT-Cog Subscales and HADS Anxiety *. Sig correlation with overall FACT Total score.
Cimprich (1999)	Correlation			Y	Sig. Inverse correlation between AFI scores and Anxiety (Total Mood Disturbance)
‌Cimprich et al. (2005)	Correlations and Regression	Y	Sig. small inverse correlations between digit span backwards test and trail making test B and POMS-SF. Increased scores on anxiety subscale were correlated with increased test performance.	Y	Anxiety sig predictor of level of perceived cognitive effectiveness. Sig strong relationship between POMS-SF scores and AFI subscales.
‌Fayette et al. (2023)	Regression	N	No relationship between objective tests and Anxiety		
Gates et al. (2024)		N	No relationship between objective tests and Anxiety		
Lange et al. (2020)	Multivariate Logistic Regression	N	No relationship between objective tests and Anxiety	N	No Sig. relationship between subjective cognitive impairment and Anxiety
Lehto (2013)	Correlations (non-parametric)			Y	Preoperatively Sig. association between increased worry and subjective CI.
Hermelink et al. (2007, 2010)	Correlations	N	No relationship between objective tests and Anxiety	Y	Pre and Post treatment, inverse Sig. correlations between subjective CI and Anxiety.
Janelsins et al. (2022)	Mixed Linear Models	Y	Baseline anxiety is associated with worse performance on some cognitive tests. Including rapid visual processing, category fluency and immediate recall*.	Y	Higher Baseline anxiety sig. Associated with PCI *
Juríčková et al. (2023)	Correlation	N	No relationship between objective tests and Anxiety		
‌Knoerl et al. (2024)	Correlations and Regression			Y	At diagnosis sig. Inverse correlation between baseline cognitive function and Anxiety.
Patel et al. (2015)	Multivariable Linear Regression	N	No relationship between objective measures and Anxiety		
‌Tulk et al. (2023)	Correlation and HMR	N	No relationship between objective measures and Anxiety	Y	Deterioration of PCI during 12 m follow-up sig. Associated with increased Anxiety.

* = Included as Supplementary Information.

#### Depression

Fourteen studies measured the relationship between levels of depression and objective cognitive impairment (Table 7). Of these, nine studies reported no relationship between depression and objective cognitive function (Aspelund et al., 2024; Cimprich and Ronis, 2001; ‌Cimprich et al., 2005; ‌Fayette et al., 2023; Gates et al., 2024; Hermelink et al., 2007; Hermelink et al., 2015; Lange et al., 2020; ‌Tulk et al., 2023). Five studies reported a relationship between at least one objective cognitive test and levels of depression (Bernstein et al., 2018; ‌Janelsins et al., 2022; Jung et al., 2022; Juríčková et al., 2023; Scheibel, 2004). Within these, based on reported domains (Table 3), affected cognitive domains included motor dexterity, verbal memory, visual memory, concentration (Bernstein et al., 2018), executive function (Bernstein et al., 2018; ‌Janelsins et al., 2022), memory (‌Janelsins et al., 2022), processing speed (‌Janelsins et al., 2022; Juríčková et al., 2023) and visual motor and sequencing skills (Scheibel, 2004). In all cases, depression was inversely related to cognitive performance. Eleven studies measured the relationship between depression and subjective cognitive impairment. Four studies reported no significant relationship (‌Knoerl et al., 2024; Lange et al., 2020; ‌Savard et al., 2023; ‌Tulk et al., 2023). The remaining seven studies reported some associations between depression and subjective cognitive impairment, relationships were inverse except for Bernstein and colleagues (Bernstein et al., 2018), who found a positive relationship between depression and two FACT-Cog subscales (Table 7). Results are summarized below in Table 7.

**Table 7:** Summary of relationships between depression and cognitive impairment.

Study	Objective cognitive impairment			Subjective cognitive impairment	
	Analysis	Significant relationship Y/N	Findings	Significant relationship Y/N	Findings
Aspelund et al. (2024)	Regression	N	No relationship between objective tests and Depression	Y	Depression was a Sig. predictor of cognitive complaints (*p* = 0.01)
Bernstein et al. (2018)	Correlations	Y	Sig. Inverse correlation between motor dexterity, verbal memory, Visual memory, processing speed, concentration, executive function,	Y	Depression mixed correlations with FACT-Cog subscales. Positive correlation with PCI subscale and overall score. Negative PCA, CFO and IQL subscales.*
Cimprich (1999)	Correlation			Y	Sig. Inverse correlation between AFI scores and (Total Mood Disturbance)
Cimprich and Ronis (2001)	Correlation and multiple regression	N	No relationship between objective tests and depression		
Cimprich et al. (2005)	Correlations and regression	N	No relationship between objective tests and depression	Y	Depression sig predictor of level of perceived cognitive effectiveness. Sig strong relationship between POMS-SF scores and AFI subscales.
‌Fayette et al. (2023)	Regression	N	No relationship between objective tests and depression		
Gates et al. (2024)		N	No relationship between objective tests and depression		
Lange et al. (2020)	Multivariate logistic regression	N	No relationship between objective tests and Depression.	N	No Sig. relationship between subjective cognitive impairment and Depression
Hermelink et al. (2007, 2010)	Correlations	N	No relationship between objective tests and Depression	Y	Sig. Inverse relationship between Depression and FEDA and EORTC-CF. Depression sig. Predictor of subjective cognition (FEDA and EORTC-CF).
Hermelink et al. (2015, 2017)	Multivariable linear regression	N	No relationship between objective tests and Depression	Y	Sig. Association of Depression on scores on self-report measures (EORTC-CF, FEDA)
Janelsins et al. (2022)	Mixed linear models	Y	Baseline depression associated with worse performance on some cognitive tests. Including Hopkins verbal learning test and Trail making test*.	Y	Higher Baseline depression sig. Associated with PCI*
Jung et al. (2022)	Regression	Y	Sig. association between lower total cognitive function and Depression.		
Juríčková et al. (2023)	Correlation	Y	Depression sig. Inverse correlation with speed of processing/psychomotor speed.		
‌Knoerl et al. (2024)	Correlations and regression			N	No/Weak relationship between depression and EORTC-CF scores.
‌Savard et al. (2023)	Repeated measures LMM			N	No relationship between depression and FACT-Cog scores.
‌ Scheibel (2004)	Correlations and analysis of variance	Y	Changes on the MMPI-D scale were related to change in scores from the TMT-B. But other correlations were not sig.		
‌Tulk et al. (2023)	Correlation and HMR	N	No relationship between objective measures and Depression	N	No relationship between Depression and Subjective cognitive impairment. (FACT-Cog)

* = Included as Supplementary Information.

### Changes in cognitive impairment across time

Sixteen studies included multiple timepoints (Table 8). Seven studies had two assessment timepoints (De Rosa et al., 2021; Gates et al., 2024; ‌Knoerl et al., 2024; Lehto, 2013; Scheibel, 2004; ‌Tulk et al., 2023; Zhuang et al., 2023). Nine studies had 3 or more assessment time points (Cimprich and Ronis, 2001; ‌Fayette et al., 2023; François et al., 2024; Hermelink et al., 2010; ‌Hermelink et al., 2017; ‌Hu et al., 2023; ‌Janelsins et al., 2022; ‌Patel et al., 2023; ‌Savard et al., 2023). For most studies, assessments included a baseline pretreatment assessment followed by a one or more post or during treatment follow up. One study (‌Knoerl et al., 2024) conducted two assessments one prior to study randomization and the final prior to surgery and did not explore changes in cognitive function over time. Nine studies assessed changes in cognitive impairment or function over time in relation to healthy control groups (Cimprich and Ronis, 2001; ‌Fayette et al., 2023; Gates et al., 2024; ‌Hermelink et al., 2017; ‌Hu et al., 2023; ‌Janelsins et al., 2022; Lehto, 2013; ‌Patel et al., 2023; Zhuang et al., 2023). Eight studies reported that between first and final assessments, changes were observed in objective cognitive function (‌Fayette et al., 2023; Gates et al., 2024; Hermelink et al., 2007; ‌Hermelink et al., 2017; ‌Janelsins et al., 2022; Scheibel, 2004; ‌Tulk et al., 2023; Zhuang et al., 2023). Eleven studies assessed changes in subjective cognitive function over time (Table 8). Of these, seven studies reported changes in subjective cognitive function overtime (De Rosa et al., 2021; Gates et al., 2024; Hermelink et al., 2010; ‌Hu et al., 2023; ‌Janelsins et al., 2022; ‌Savard et al., 2023; ‌Tulk et al., 2023). For objective cognitive function results were varied. Results are summarized below in Table 8.

**Table 8:** Summary of changes in cognition.

Study details				Objective cognitive impairment		Subjective cognitive impairment	
Study	HCs Y/N	Timepoints	Timepoint details	Change in cognition overtime	Findings	Change in cognition overtime	Findings
Cimprich and Ronis (2001)	Y	3	1. Before Surgery 2. Approx. 2 Weeks post-surgery 3. Approx. 3 months post-surgery	N	Non. Sig trend of change over time for total attention scores. The mean TAS score at T3 was sig. Improved compared to T1 (*p* = 0.16) For the patient group, there was a significant group x time interaction (*p* = 0.005) breast cancer group gradual gain in Total Attention Score cognitive function overtime.		
De Rosa et al. (2021)	N	2	1. Before starting surgical and or medical treatment 2. 6 months from the end of therapy			Y	Sig. reduction in PCI scores from T1-T2 (*p* < 0.0.05).
‌Fayette et al. (2023)	Y	3	1. Prior to treatment 2. Promptly after treatment 3. 12 months after baseline assessment	Y	Sig. Group differences pre and post chemotherapy. Deficits also persisted at third assessment verbal memory and learning, and abstraction/executive function. Sig improvement across both groups between 1–3 timepoints across some cognitive domains but still remained less than HCs.		
‌François et al. (2024)	N	5	1. Inclusion 2. Prior to surgery 3. 3 months post 4. 6 months post 5 0.12 months post	N	No. sig differences in MMSE scores across any timepoints.	N	No sig improvements at a patient level overtime. Some between group differences sig. at 3 M Overall gradual EORTC non. Sig improvement.
Gates et al. (2024)	Y	2	1. Treatment naive baseline assessment 2. 6–8 weeks post treatment assessment	Y	Fixed effect for time for HVLT-R (p < 0.001) and Delayed recall (*p* = 0.046) with sig. Improvements post treatment for patients.	Y	Patients perceived cognitive impairment and perceived cognitive abilities were worse at follow up compared to baseline (*p* = 0.004).
Hermelink et al. (2007, 2010)	N	3	1. Before the start of preoperative chemotherapy (T1) 2. Before the last chemotherapy cycle (approx. 5 m later) (T2) 3. Approx 1 year after baseline (T3)	Y	At T2 MANOVA sig. Overall improvement in tests (p < 0.001). Cognitive decline observed in 27% of patients and improvement in 28% of patients.	Y	Significant increase in cog problems from T1-T2, significant increase in cognitive problems for both FEDA and EORTC-CF (Both p < 0.001), partial recovery at T3.
Hermelink et al. (2017)	Y	3	1. Prior to primary surgery or neoadjuvant chemotherapy 2. A min of 1 week after completion of chemotherapy 3. One year after T1	Y	Decline in total cognitive indices scores in both patient groups relative to controls (*p* = 0.04). Steady improvement was observed in the sample as a whole.		
‌Hu et al. (2023)	Y	3	1. Before neoadjuvant chemotherapy 2. Before the second cycle of neoadjuvant chemotherapy 3. Completion of neoadjuvant chemotherapy	N	No sig changes in neuropsychological test scores.	Y	Compared with prior to neoadjuvant chemotherapy, the scores on the FACT-Cog declined significantly at assessment 1 and 2, and scores on the PCA subscale declined at assessment 2 (p < 0.05).
Janelsins et al. (2022)	Y	3	1. T1 Prechemotherapy -within 7 days prior to the first chemo 2. A2 post chemotherapy within 1 month of the last chemo administration 3. A3 6-month follow-up from A2	Y	Between the first and third assessment, patients’ performance was worse than controls and showed less improvement over time. Across multiple tests.	Y	Across time patients reported more cognitive complaints compared with controls including attention difficulty (*p* = 0.01) and perceived cognitive impairment (*p* < 0.05).
‌Knoerl et al. (2024)	N	2	1. Prior to randomization 2. Prior to Surgery			N/A	Secondary analysis, did not explore change in cognition, only in relation to anxiety levels.
Lehto (2013)	Y	2	1. Time of treatment planning following diagnosis 2. 3–4 weeks after surgical resection			N	Between timepoints, malignancy group did not drop significantly in cognitive effectiveness on the AFI.
Patel et al. (2023)	Y	4	1. Baseline before any systemic or local treatment 2. Approx 1 month after completion of their primary cancer treatment. 3. 1 year after treatment completion 4. 2 years after treatment completion	N	Objective test scores were unchanged from baseline in patients but showed modest improvement in controls.	N	Levels of subjective cognitive function from baseline were unchanged for patient groups. Controls showed modest improvement.
Scheibel (2004)	N	2	1. Baseline 2. On - treatment	Y	Main effect of time for digit symbol (*p* < 0.027) (although will within normative) CLTR effect of time (*p* < 0.021) TMT-B effect of time (*p* < 0.009), Declines on treatment.		
‌Savard et al. (2023)	N	5	1. Baseline 2. 3 months post 3. 6 m post 4. 9 months 5. 12 months			Y	Sig, reductions in Fact-Cog Impact scores overtime (*p* < 0.001)
‌Tulk et al. (2023)	N	2	1. Before beginning prostate cancer treatment (baseline) 2. 12 months following first evaluation	Y	Patients had sig. Declines over first year of treatment when compared with group with no cognitive function issues at baseline.	Y	29% of patients demonstrated sig. Declines in subjective cognition during first year of treatment.
Zhuang et al. (2023)	Y	2	1. T0 before neoadjuvant treatment or surgery 2. T1 1 week after completing chemotherapy (approx. 5–6 months post)	Y	Sig. lower performance in the LTT and WDT tests post chemotherapy (*p* = 0.002, *p* = 0.003) for patients.		

## Discussion

To our knowledge, this is the first review to synthesize levels of pretreatment subjective and objective cancer related cognitive impairment, across multiple cancer types and treatments and its relationship with both anxiety and depression. Secondary objectives included investigating associations between objectively measured and subjectively reported cognitive issues. Finally, longitudinal changes across time into the postoperative period were explored. Pretreatment CRCI was evident across studies, particularly subjective CRCI, and inclusion of healthy control groups allowed for increased interpretation of CRCI levels. Support was also found for a relationship between psychological variables and subjective cognitive function, particularly with regards to anxiety. Heterogeneity across study designs prevented further analysis of the trajectory of change in cognition across time from pre to post treatment.

Heterogeneity and variance were aspects of both study characteristics and outcomes of interest within this review. The heterogeneity in subjective measures of cognitive impairment used presents some difficulty in interpretation given the differences between common assessment tools. As reviewed by the Cancer Neuroscience Initiative Working group in 2021 (Henneghan et al., 2021), the EORTC-CF, which was used in 6 papers, consists of only 2 items, and may not be adequate to fully capture the complex nature of CRCI assessment, as opposed to the FACT-Cog which consists of 37 items and 4 subscales, was used in 9 papers. Although single item measures can report cognitive change among cancer patients during chemotherapy (Onyedibe et al., 2025), levels of subjective CRCI may be over or underreported when relying on a shorter measure. Variance was also noted in the objective cognitive assessments, used, but in general studies were consistent in investigating common domains such as attention, memory and executive function. This is possibly a consequence of the International Cancer and Cognition Task Force, which recommended in 2011 specific cognitive tests and domains of interest to standardize objective CRCI research (Wefel et al., 2011). Notably, the three primary tests they recommended were all represented in the most commonly used tests within the current review, including the most used test, the trail-making test. This indicates that objective CRCI research is largely adhering to standardized approach to assessing recommended domains which will allow for increased comparison across studies as the evidence base grows.

The review findings indicate that levels of pretreatment CRCI remain a complex topic, and that more targeted research at the pretreatment timepoint is required to fully understand levels of CRCI pretreatment. Research is needed to further establish pretreatment CRCI causes and associations within research, across cancer types beyond breast, to understand why it can occur. Compared to Hutterer and Oberndorfer (2021) review paper exploring CRCI prevalence across timepoints, levels of subjective CRCI pretreatment observed in this review were comparable to that observed prechemotherapy only. In contrast, levels of objective CRCI observed were higher. As this review focused on pre any treatment or surgery, these comparisons shed light on the possible existence of pretreatment CRCI from diagnosis or prior any surgery or treatment. This review further builds on this research by observing largely similar levels of CRCI prior to both chemotherapy and surgery. It also highlights the importance of understanding pretreatment CRCI at a cancer specific level and identifying potentially at-risk cohorts. The study that recorded the highest level of pretreatment objective impairment (Jung et al., 2022), pertained to newly diagnosed patients with thyroid cancer. As we know that hormonal treatment effects have been explored in CRCI modelling as impacting on function (Hutterer and Oberndorfer, 2021), there is an increased need to investigate pretreatment CRCI in cancers that may be more susceptible to pretreatment CRCI.

Compared to healthy control groups, increased subjective and objective impairment was evident pretreatment. Objective function was more varied, including instances of increased performance of patient groups relative to healthy controls in some cases. These findings speak to the importance of including a healthy control group for test comparison as they can provide insight beyond standardized test or population norms, which may not capture the mild to moderate CRCI experience.

With regard to the relationships between objective and subjective cognitive level of impairment only seven studies directly reported on this relationship. Of these, only two reported any association between objective test performance and self-reported subjective cognitive function. This lack of research was notable, as the relationship between self-reported cognitive impairment, and objective cognitive performance is a keen area of interest within CRCI research (Ahles and Hurria, 2018). A lack of association between objective and subjective CRCI has been previously noted within the literature (Fardell et al., 2022; Hutchinson et al., 2012), potentially due to insufficient sensitivity of neuropsychological tests for CRCI, or associations between subjective CRCI and other psychological factors (Ahles and Hurria, 2018; Zhang et al., 2025). Differences in assessment administration, assessment timeframes and the misattribution of cognitive difficulties when applying self-report tools could help further explain these discrepancies (Hutchinson et al., 2012). As patients with cancer can have high indices of anxiety and depression, emotional state during test or self-report administration may also be impacting both test scores and perceived impairment (Hutchinson et al., 2012; Zhang et al., 2025). More research investigating the relationships between objective and subjective CRCI will aid in our understanding of CRCI experiences, its association with other variables like psychological wellbeing, and how to help patients experiencing these issues. Other areas of research such as cognitive aging could give us more insight into the temporal relationships between objective and subjective cognitive function (Snitz et al., 2015) and inform future research.

It was clear from results that anxiety and depression had some impact on CRCI, however the nature of these relationships varied across both psychological and cognitive variables. Little support was found for a relationship between anxiety and objective cognitive function. In contrast, only two studies that assessed anxiety and subjective cognitive function reported no relationship. These findings speak to the complex nature of CRCI, and the potential psychological underpinnings. Pretreatment subjective cognitive experience may better align conceptually with the overall psychological burden of cancer diagnosis, as factors like pretreatment worry and anxiety could possibly influence perception of cognition, as both experience of anxiety and perception of cognition are recorded using self-report measures and as such are influenced by the patient’s own perceptual experiences. This idea has previously been explored in recent models like Oppegaard and colleagues (Oppegaard et al., 2023) multifactorial models of CRCI. When this model was evaluated within cancer patients during treatment, they found that co-occurring symptoms like state anxiety produced the largest variance in subjective cognitive function (Oppegaard et al., 2023b). Results also reflect other conceptual CRCI frameworks, by highlighting associations between CRCI and psychological variables. Some models suggest that sociodemographic and psychological factors may act alongside treatment related factors to increase likelihood of post treatment cognitive impairment (Ahles and Hurria, 2018). The present review provides further insight into the complexity of these interactions, observing the presence of relationships between psychological variables and CRCI, prior to an interaction with treatment related effects. Results related to depression were more mixed. Compared to anxiety, more support was found for relationships between subjective cognitive impairment and depression compared to objective cognitive function with seven of 11 studies reporting a significant association. Findings indicate overall that subjective cognitive impairments are associated with psychological factors like anxiety and depression more than objective measures. This review extends prior work exploring psychological variables and CRCI through breast cancer (Yang and Hendrix, 2018), by expanding across cancer types, also suggesting an influence of psychological variables on CRCI. Understanding these relationships is critical for the future of CRCI treatment intervention providing individualized patient psychological support.

Changes in cognition across time were explored in studies with multiple assessment timepoints. However, conclusions that could be drawn were limited due to heterogeneity across both number of assessment timepoints and duration of assessment periods. Results of objective change were varied and included both improvements and declines in performance across timepoints. For subjective CRCI, results were more consistent and indicated overall increases in subjective cognitive impairment over time. Standardization of assessment timepoints and inclusion of a core set of patient reported outcomes like psychological wellbeing when investigating CRCI could help to elucidate the long-term relationships between cognitive change and associated variables. This will in turn aid in the creation of appropriate long-term treatment and support for survivors.

There are some limitations of the present review. Searches were limited to those only published in English. Many of the studies also employed convenience sampling through specific clinics and hospitals, limiting generalizability. Furthermore, the majority of papers excluded patients based on presence of metastasis, CNS involvement, prior history of cancer or treatment, or included specific cancer/tumor stages. This was important to ascertain given the focus of the present review on pretreatment cognitive impairment, as presence of a historical cancer diagnosis or treatment makes it difficult to fully extrapolate whether baseline CRCI levels reported are a true reflection of pretreatment CRCI.

Although inclusion criteria were not based on cancer type, 40% of included studies pertained to breast cancer cohorts, where the age at diagnosis is typically around the fifties (Hendrick et al., 2021). The representation of breast cancer within this review is reflective of the current state of CRCI literature, which traditionally focused on breast cancer patients and chemotherapy induced changes (Ahles and Hurria, 2018; Ahles and Saykin, 2007).

Insights from this review can help to inform future CRCI research. CRCI research would benefit from increased rigor when applying objective and subjective cognitive measures and patient reported outcomes to allow for more nuanced comparisons and analysis within this research landscape. By increasing standardization of measures this will allow for increased comparison across studies. Although patterns and common neuropsychological tests emerged, there was still a large volume of different tests applied across studies (Table 3). Also of note was the marked contrast in terms of item count between longer measures like the FACT-Cog, and shorter measures such as the EORTC-CF scale (Henneghan et al., 2021). By standardizing common objective and subjective tools, as well as recording psychological wellbeing as standard, this will allow for a fuller picture of the relationship between CRCI and psychological variables. Insight can also be gained from work conducted around cognitive perception during aging (Snitz et al., 2015). On multiple occasions, the presence of a healthy control group allowed for a richer interpretation of research findings and shed light on the complexity of CRCI research. Where possible, including healthy control groups will provide increased insight and the opportunity to elucidate how CRCI may present. Inclusion of both baseline testing timepoints, and detail of prior and current treatment history will allow for increased rigor when investigating pretreatment CRCI. Increasing standardization across time points within longitudinal research will also provide the opportunity for generalizability, comparison across studies, and a holistic interpretation of CRCI from diagnosis to survivorship. By assessing cognitive impairment as early as possible, we can learn more about its trajectory and the interaction with treatment effects, including not only chemotherapy or radiotherapy, but also surgical effects like anesthesia exposure, particularly in elderly populations (Sun et al., 2019). We can also learn smore about potential prehabilitative or rehabilitative interventions to target CRCI and psychological wellbeing (Levett and Grimmett, 2019; Scriney et al., 2022). Review findings suggested that there may be a relationship between psychological variables and subjective cognitive impairment so efforts should be made to assess both in future CRCI research. Potential future research could also focus on addressing both subjective CRCI experience, and psychological difficulties through targeted interventions.

## Conclusion

This review explored pretreatment CRCI and its association with psychological variables across cancer types and treatments. Some support was found for associations between subjective cognitive function and psychological variables, in particular anxiety. Associations between psychological variables and objective CRCI were largely inconsistent. Findings indicated weak associations between objective and subjective measures of cognitive function. Variation in study design prevented further analysis of trajectory of change, but some declines in subjective cognitive function were observed. This review highlighted the heterogeneity that currently exists in the CRCI landscape, and that increased standardization would benefit future research. Understanding more about pretreatment CRCI, and its relationship with psychological variables will benefit clinicians and other allied health professionals when caring for patients and will also provide further insights for the application of pre and rehabilitation care. This review highlights the complex relationship between cognitive function and psychological wellbeing and the need to future understand this relationship to ultimately enhance patient wellbeing and pre and postoperative cognitive care.

## Data Availability

The original contributions presented in the study are included in the article/Supplementary material, further inquiries can be directed to the corresponding author/s.
